# Disease burden and health-related quality of life among adults aged 50 years or older with herpes zoster in Huzhou, China

**DOI:** 10.3389/fpubh.2026.1929296

**Published:** 2026-09-09

**Authors:** Shangyan Han, Jie Gong, Jianyong Shen, Xiaofu Luo, Zizhe Zhang, Xiaoqi Liu, Yu Hu

**Affiliations:** 1 School of Public Health, Hangzhou Medical College, Hangzhou, Zhejiang, China; 2 Institute of Immunization, Zhejiang Provincial Center for Disease Control and Prevention, Hangzhou, China; 3 Institute for Health Policy, Hangzhou Medical College, Hangzhou, Zhejiang, China; 4 Institute of Immunization, Huzhou Center for Disease Control and Prevention, Huzhou, China

**Keywords:** disease burden, EQ-5D-5L, EQ-VAS, health-related quality of life, HZ, older adults, PHN, vaccine

## Abstract

**Background:**

This study assessed the disease burden and health-related quality of life (HRQoL) among adults aged 50 years and older with herpes zoster (HZ) in Huzhou, China, and identified factors associated with increased disease burden.

**Methods:**

A cross-sectional study was conducted among adults aged 50 years or older who developed HZ between 1 June 2024 and 1 June 2025 in Huzhou, China. Participants completed a structured questionnaire in September 2025 after the acute phase and retrospectively reported HRQoL during the acute HZ phase; participants with PHN also reported HRQoL during the PHN phase. Disease-burden and HRQoL distributions were compared using the Mann–Whitney U test or Kruskal–Wallis test, as appropriate. Gamma generalized linear models (GLMs) with a log link were used to identify factors associated with total, direct, and indirect burden.

**Results:**

A total of 222 participants were included, of whom 62 (27.9%) developed PHN. Costs were recorded in RMB as gross disease-related expenditures before reimbursement and converted using USD = RMB/7.1. The median direct burden was RMB 39,998.58 (P25, P75: RMB 11,792.27, 277,962.43), equivalent to USD 5,633.62 (P25, P75: USD 1,660.88, 39,149.64), and the median indirect burden was RMB 1,843.59 (USD 259.66; P25, P75: USD 155.79, 519.31). Participants with PHN had a higher median total burden than those without PHN (USD 10,958.43 vs. USD 4,740.60; *p* < 0.001). During the acute HZ phase, the median EQ-5D-5L utility and EQ-VAS scores were 0.40 (P25, P75: −0.03, 0.53) and 10.00 (P25, P75: 10.00, 10.00), respectively. Treatment setting, PHN status, and age were associated with disease burden.

**Conclusion:**

Herpes zoster was associated with substantial disease burden and markedly impaired HRQoL among adults aged 50 years or older in Huzhou. PHN and inpatient treatment identified subgroups with particularly high burden. These regional estimates may inform prevention policy and future evaluations of HZ vaccination.

## Introduction

Herpes zoster (HZ), commonly referred to as shingles, is caused by the reactivation of latent varicella-zoster virus (VZV) (1). The incidence of HZ increases with age due to the progressive decline in cell-mediated immunity (2–4). As populations continue to age, the disease burden associated with HZ is expected to increase, making it an important public health concern (2, 5).

PHN is the most common complication of HZ and is characterized by persistent pain that continues after rash healing (6, 7). The pain may last for months or years and can substantially impair physical functioning and quality of life (6, 8, 9). PHN is also associated with increased healthcare utilization and higher medical costs (10, 11). The risk of HZ and its complications begins to rise from approximately 50 years of age and increases further with advancing age as cell-mediated immunity declines (2, 4, 6, 12). Accordingly, age 50 years is a clinically and policy-relevant threshold for this study population.

The HZ and PHN are associated with a substantial economic and health burden (10, 11, 13–15). Direct costs mainly include outpatient visits, hospitalization, and medication use, while indirect costs arise from productivity loss and caregiver burden (10, 11, 16). Both conditions can also substantially reduce HRQoL (8, 9, 17–19).

Several studies have examined the burden of HZ across different countries. In Spain, the mean societal cost was €296 per HZ case and €712 per PHN case. EQ-5D utility decreased by 0.138 during the first month after diagnosis (20). In Japan, the mean total cost per patient with HZ was ¥57,112, of which 77% was attributable to direct medical costs (21). In Germany, the annual societal cost of HZ and PHN was estimated at €182 million (22).

Evidence from China remains limited (12, 13). Li et al. (13) reported a mean HZ-related cost of RMB 840.6 per patient and a mean hospitalization cost of RMB 4,502.4. A recent prospective study in China also showed that both HZ and PHN substantially impaired quality of life, with PHN causing more severe and longer-lasting reductions than HZ alone (17). Together, these findings suggest that HZ and PHN impose considerable economic and health burdens on affected individuals in China.

Recombinant zoster vaccine (RZV) is approved or recommended for adults aged 50 years or older in many settings. Observational evidence has associated zoster vaccination with a lower risk of HZ in older adults with selected immune-mediated diseases (23). Public-health recommendations have long identified zoster prevention in older adults as an important priority (24). Because the risk of HZ rises from around this age, local estimates of disease burden can help frame prevention priorities and guide subsequent policy and economic evaluations.

Although several studies have evaluated the burden of HZ in China, most have focused on healthcare costs alone (12, 13). Few studies have assessed direct costs, indirect costs, HRQoL, and PHN-related outcomes simultaneously within the same population (17, 18, 25). Evidence from eastern China remains scarce.

Huzhou is a rapidly developing city in Zhejiang Province and provides a suitable setting for assessing the burden of HZ among older adults in eastern China. This study aimed to quantify direct, indirect, and total disease burden and describe HRQoL among adults aged 50 years or older with HZ in Huzhou, and to identify factors associated with higher burden. The findings may inform prevention policy and future vaccine evaluations but do not directly evaluate vaccine effectiveness or cost-effectiveness.

## Methods

### Study design and participants

We conducted a cross-sectional study in Huzhou, Zhejiang Province, China. The survey was conducted between 1 September and 30 September 2025. Eligible patients had developed HZ between 1 June 2024 and 1 June 2025 and had completed the acute HZ phase, with clinical resolution of the rash confirmed by the time of the survey. The median interval from HZ rash onset to questionnaire completion was 289 days (P25, P75: 198, 381 days). HZ cases were identified from the Huzhou Health Management Database using the International Classification of Diseases, Tenth Revision (ICD-10) code B02.901. PHN was clinically defined as HZ-related pain persisting for at least 90 days after rash onset. PHN status was initially ascertained using the recorded ICD-10 code G58.001 and was subsequently confirmed through a review of clinical records and the structured patient interview. Because all participants developed HZ no later than 1 June 2025 and were surveyed no earlier than 1 September 2025, every participant had an observation period of at least 92 days after rash onset. Participants were classified as not having PHN only when neither the clinical records nor the interview documented HZ-related pain persisting at or beyond 90 days after rash onset.

Patients were eligible if they met the following criteria: (1) development of HZ between 1 June 2024 and 1 June 2025; (2) age 50 years or older; and (3) completion of the acute disease phase at the time of survey. Patients were excluded if key clinical or economic data were missing, they had severe cognitive impairment or communication difficulties, or they declined participation. All eligible records identified during the prespecified period were approached; therefore, recruitment used a census of the available database cohort rather than an *a priori* sample-size target.

### Data collection

We reviewed published studies on the disease burden and HRQoL associated with HZ and PHN to inform questionnaire development (17, 26). The questionnaire was developed with reference to previously published HZ burden studies and validated HRQoL instruments, including the EQ-5D and EQ-VAS (13, 17, 27, 28). The questionnaire included demographic characteristics, clinical information, healthcare resource utilization, direct and indirect costs, and HRQoL measures. Three public health researchers and two health economists reviewed the questionnaire content. A pilot survey involving 15 older adults was conducted before formal data collection to assess clarity and feasibility. The final questionnaire was reviewed by experts in public health, epidemiology, and health economics before implementation.

The questionnaire included seven sections: (1) sociodemographic characteristics; (2) clinical characteristics and disease management; (3) direct medical and non-medical costs, including gross amounts charged for outpatient care, hospitalization, and medicines before reimbursement, together with patient-paid transportation and health products; (4) HZ-related days lost from paid work or usual productive activities; (5) retrospectively reported HRQoL during the acute HZ phase and, for participants with PHN, the PHN phase; (6) history of HZ vaccination; and (7) chronic comorbidities.

Only costs attributed to the index HZ episode were recorded; unrelated medical expenditures were excluded. Direct medical costs were verified by comparing participant recall with records from the Huzhou Health Management Database. Vaccination history was confirmed through the Zhejiang Provincial Immunization Information System.

### Disease burden assessment

Disease burden was assessed from a limited societal perspective, combining total HZ-attributable medical expenditures regardless of payer, patient-paid direct non-medical costs, and patient productivity losses. Direct medical costs were recorded in nominal RMB as the total amounts charged for HZ-related outpatient care, hospitalization, and medicines before any medical-insurance or other reimbursement; they therefore represent gross billed expenditures rather than net patient out-of-pocket payments. Direct non-medical costs comprised patient-paid transportation and health products. These categories were recorded separately, and their component-level distributions are presented in Supplementary Table S1. Direct burden was calculated as direct medical costs plus direct non-medical costs. Indirect burden was estimated using the human capital approach (29, 30) as HZ-related days lost from paid work or usual productive activities multiplied by RMB 184.356 (USD 25.966) per day. The daily value was calculated from the 2024 annual per capita disposable income of Huzhou residents (RMB 67,290) divided by 365 days (31). The same daily value was applied to employed, retired, unemployed, self-employed, and other non-salaried participants; for participants without salaried employment, it represents the opportunity cost of lost time rather than actual wage loss. Informal-caregiver productivity loss and payer administrative costs were excluded. Total burden equaled direct burden plus indirect burden. RMB amounts were converted to USD using the rounded rate USD 1 = RMB 7.1, based on the 2024 monthly period-average RMB/USD series published by the People’s Bank of China (32).

### Health-related quality-of-life assessment

The HRQoL was assessed retrospectively after the acute HZ phase using the validated Chinese EQ-5D-5L and EQ-VAS instruments (27, 33). All participants recalled their health during the acute HZ phase; the 62 participants with PHN also recalled their health during the PHN phase. Because these phase-specific estimates were derived from different analytic populations, they were not interpreted as a within-person comparison.

The EQ-5D-5L measures health status across five dimensions: mobility, self-care, usual activities, pain/discomfort, and anxiety/depression. Utility scores were calculated using the Chinese EQ-5D-5L value set (33). Scores range from −0.391 to 1.000, with higher scores indicating better health status.

The EQ-VAS measures self-rated health on a scale from 0 to 100. Higher scores indicate better perceived health.

### Statistical analysis

All analyses were performed using IBM SPSS Statistics, version 31.0.

Continuous variables were summarized as mean (SD) or median (P25, P75), according to their distributions. Categorical variables were summarized as frequencies and percentages.

Disease-burden outcomes were non-normally distributed and are presented as median (P25, P75). Two-group comparisons were performed using the Mann–Whitney *U* test, and comparisons across three or more groups were performed using the Kruskal–Wallis test. Because EQ-5D-5L utility scores and EQ-VAS scores are bounded and showed non-normal distributions, HRQoL outcomes are also presented as median (P25, P75). Differences in HRQoL across age groups were assessed using the Kruskal–Wallis test. All tests were two-sided, and *p* < 0.05 was considered statistically significant.

All disease-burden outcomes were positively skewed and strictly positive; no zero-cost observations occurred. Separate Gamma GLMs with a log link were fitted for total, direct, and indirect burden (34, 35). Gender, treatment setting, age, chronic comorbidity, rash distribution, rash duration, and PHN status were entered simultaneously without stepwise selection, with gender retained regardless of statistical significance. In the total- and direct-burden models, age group was entered as a single ordinal variable coded 1 = ≥80 years, 2 = 70–79 years, 3 = 60–69 years, and 4 = 50–59 years; its exponentiated coefficient represents the multiplicative change in expected burden for each one-category step toward a younger age group. In the indirect-burden model, age was entered as a continuous variable in years. Model fit was assessed using deviance/df (total:1.08, direct:1.05, indirect:0.96) and Pearson *χ*^2^/df (total: 1.12, direct: 1.09, indirect: 1.03).

For categorical contrasts, the reported exponentiated coefficients compared female with male, outpatient with inpatient treatment, no with yes for chronic comorbidity, single with multiple dermatomes, rash duration of at least 2 weeks with less than 2 weeks, and no PHN with PHN. All statistical tests were two-sided, and *p* < 0.05 was considered statistically significant.

### Ethics approval and consent to participate

The study was conducted in accordance with the Declaration of Helsinki and was approved by the Ethics Review Committee of the Zhejiang Provincial Center for Disease Control and Prevention (approval No. SQ-2025-033; 8 January 2025). Formal survey recruitment and questionnaire administration began only after ethics approval. Written informed consent was obtained from all participants before data collection.

## Results

### Participant characteristics

A total of 420 patients with HZ were identified from the Huzhou Health Management Database. Of these, 115 declined initial participation and 305 agreed to participate. Of the latter, 34 subsequently declined participation and 49 had incomplete questionnaires or missing key data; 222 completed valid questionnaires and were included in the final analysis (52.9% of identified records and 72.8% of those who initially agreed; Figure 1). Individual-level demographic and clinical data for the 198 non-included patients were not available for the present analysis. Therefore, a formal comparison of baseline characteristics between included and non-included patients could not be performed.

**Figure 1:**
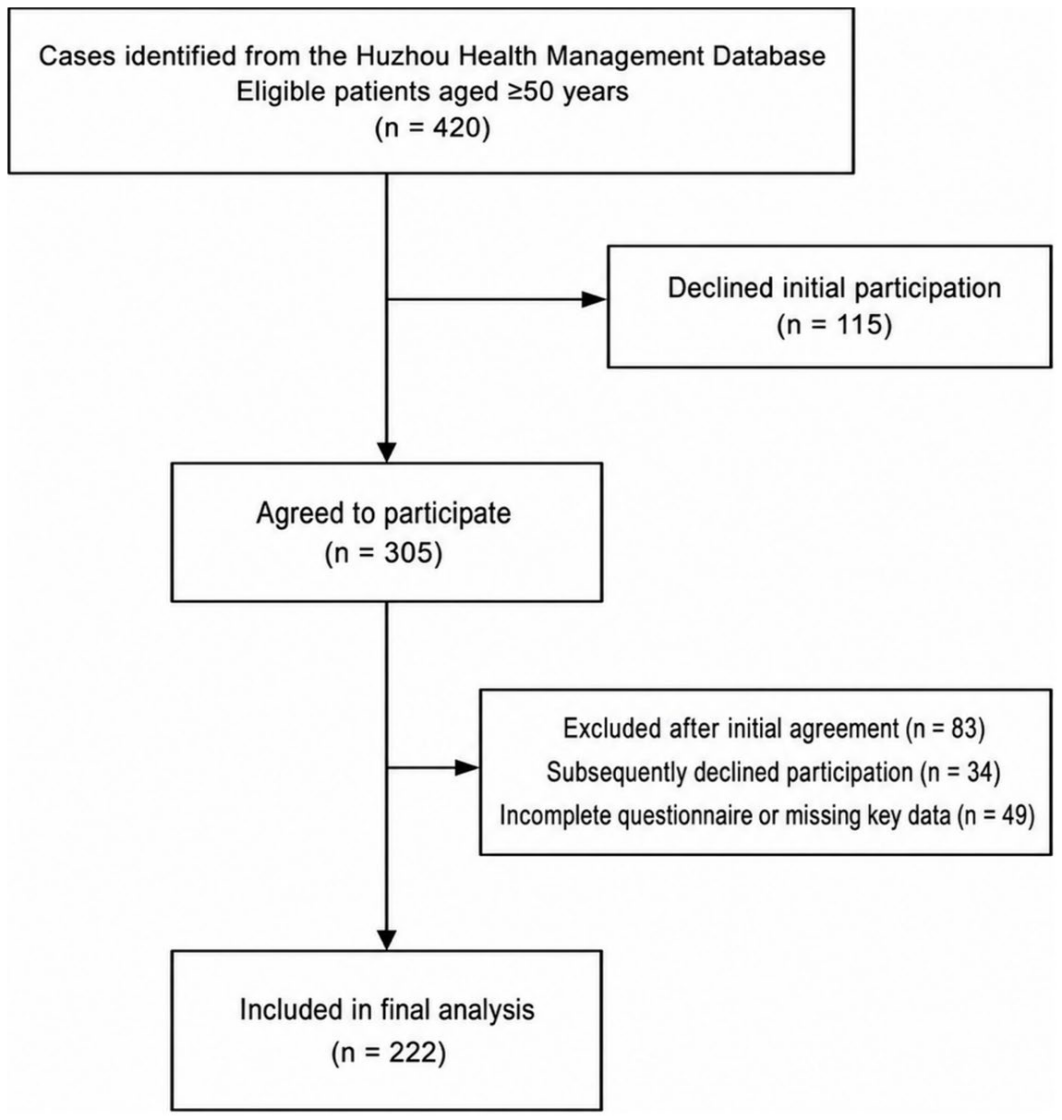
Study flowchart.

The mean age of the participants was 64.23 (SD 11.09) years, and 110 (49.55%) were male. Overall, 62 participants (27.93%) developed PHN. Seventy participants (31.53%) received inpatient treatment, whereas 152 (68.47%) received outpatient treatment. Chronic comorbidities were present in 83 participants (37.39%). Rash involvement at multiple sites was reported by 108 participants (48.65%), and 152 (68.47%) had a rash duration of more than 2 weeks (Table 1).

**Table 1:** Disease burden among adults with herpes zoster, by demographic and clinical characteristics.

Variable	Participants, *n* (%)	Direct burden (USD), median (P25, P75)	*P*	Indirect burden (USD), median (P25, P75)	*P*	Total burden (USD), median (P25, P75)	*P*
Overall	222 (100.00)	5,633.62 (1,660.88, 39,149.64)		259.66 (155.79, 519.31)		5,726.78 (1,817.29, 22,980.57)	
Gender			0.620		0.752		0.363
Male	110 (49.55)	6,587.82 (2,495.56, 20,600.99)		257.84 (154.70, 515.68)		6,817.65 (2,657.85, 21,081.35)	
Female	112 (50.45)	5,673.92 (1,250.32, 25,026.44)		256.84 (153.54, 513.72)		5,304.65 (1,483.80, 25,188.73)	
Treatment setting			<0.001		<0.001		<0.001
Outpatient	152 (68.47)	3,328.38 (979.54, 6,873.59)		180.49 (103.14, 386.76)		3,497.08 (1,264.95, 7,060.45)	
Inpatient	70 (31.53)	44,501.41 (13,344.19, 63,646.58)		515.68 (360.98, 625.26)		44,994.76 (13,597.36, 64,113.97)	
Age Group (years)			<0.001		<0.001		<0.001
50–59	58 (26.13)	2,828.10 (671.06, 8,571.69)		181.76 (149.30, 389.48)		2,996.88 (821.33, 9,091.00)	
60–69	63 (28.38)	5,097.89 (1,180.14, 41,088.03)		259.66 (129.83, 519.31)		5,474.15 (1,569.63, 41,451.55)	
70–79	50 (22.52)	6,519.15 (3,611.34, 29,865.81)		376.50 (155.79, 558.26)		6,713.90 (3,785.41, 30,884.96)	
≥80	51 (22.97)	11,566.06 (2,076.76, 25,628.73)		464.11 (180.49, 666.48)		12,044.87 (2,258.52, 25,899.22)	
Occupation			0.154		0.173		0.129
Civil Servant/Public Sector	49 (22.07)	4,342.54 (1,059.23, 37,278.31)		360.98 (180.49, 515.68)		5,225.56 (1,344.85, 37,654.81)	
Corporate/Private Sector	47 (21.17)	3,691.55 (938.31, 8,102.25)		180.49 (77.35, 360.98)		4,081.03 (1,197.97, 8,180.15)	
Farmer	36 (16.22)	7,048.17 (2,485.56, 29,810.92)		386.76 (154.70, 541.47)		8,003.23 (2,654.34, 30,161.45)	
Unemployed	45 (20.27)	5,117.89 (1,237.32, 35,698.45)		360.98 (180.49, 528.57)		5,390.53 (1,607.33, 36,272.08)	
Others	45 (20.27)	7,087.55 (2,316.27, 52,528.53)		257.84 (141.81, 515.68)		10,758.08 (2,747.90, 36,113.90)	
Education Level			0.510		0.230		0.160
≤High middle-school and Below	86 (38.74)	6,667.11 (2,686.44, 39,434.82)		257.84 (148.26, 406.10)		5,446.54 (1,725.94, 15,969.82)	
College	84 (37.84)	4,695.70 (826.51, 36,310.46)		360.98 (161.15, 515.68)		6,783.96 (2,810.27, 39,856.77)	
Postgraduate	52 (23.42)	5,215.42 (1,328.84, 15,424.54)		309.41 (161.15, 502.79)		5,542.08 (868.01, 36,544.15)	
Chronic Comorbidities			<0.001		<0.001		<0.001
Yes	83 (37.39)	9,288.31 (3,554.65, 51,527.18)		363.52 (155.79, 545.28)		9,828.67 (3,710.44, 51,890.70)	
No	139 (62.61)	4,293.52 (1,190.00, 11,785.21)		259.66 (155.79, 467.38)		4,838.80 (1,483.42, 12,332.16)	
Rash Distribution			0.001		0.008		<0.001
Single Dermatome	114 (51.35)	4,294.15 (1,097.36, 9,881.41)		257.84 (122.47, 464.11)		4,553.34 (1,376.03, 10,378.17)	
Multiple Dermatomes	108 (48.65)	9,762.25 (2,686.44, 43,079.51)		360.98 (180.49, 515.68)		10,268.58 (2,810.27, 43,443.03)	
Rash Duration			0.095		0.079		0.080
<2 weeks	70 (31.53)	4,753.10 (1,179.33, 22,420.42)		257.84 (103.14, 477.01)		5,098.31 (1,440.74, 22,900.79)	
≥2 weeks	152 (68.47)	6,645.00 (2,000.18, 23,108.56)		335.19 (154.70, 515.68)		6,813.78 (2,136.33, 23,313.64)	
PHN Status			0.010		<0.001		<0.001
Yes	62 (27.93)	10,822.11 (3,925.63, 33,521.87)		386.76 (257.84, 773.52)		10,958.43 (4,217.75, 33,963.28)	
No	160 (72.07)	4,348.94 (1,250.32, 16,468.63)		232.06 (135.37, 386.76)		4,740.60 (1,506.12, 16,897.06)	

Median (P25, P75). USD = RMB/7.1. Direct medical costs are gross HZ-attributable amounts billed before reimbursement, whereas direct non-medical costs are patient-paid; direct = medical + non-medical and total = direct + indirect. Mann–Whitney U: two groups; Kruskal–Wallis: three or more groups. HZ, herpes zoster; PHN, postherpetic neuralgia.

### Disease burden of herpes zoster

The median direct burden was RMB 39,998.58 (P25, P75: RMB 11,792.27, 277,962.43), equivalent to USD 5,633.62 (P25, P75: USD 1,660.88, 39,149.64). The median indirect burden was RMB 1,843.59 (P25, P75: RMB 1,106.11, 3,687.10), equivalent to USD 259.66 (P25, P75: USD 155.79, 519.31). Direct medical costs are gross HZ-attributable amounts billed before reimbursement, not net patient out-of-pocket payments.

Participants with PHN had a higher burden than those without PHN. Median direct burden was USD 10,822.11 among participants with PHN and USD 4,348.94 among those without PHN. Median indirect and total burdens were also higher among participants with PHN (Table 1).

Participants who received inpatient treatment had higher direct, indirect, and total burdens than outpatients. The median HZ-attributable direct burden was USD 44,501.41 (RMB 315,960.01 before reimbursement) for inpatients and USD 3,328.38 (RMB 23,631.50 before reimbursement) for outpatients. These estimates were restricted to costs attributed to the index HZ episode and exclude unrelated medical expenditures. Burden also differed by age group, chronic comorbidity, and rash distribution in unadjusted comparisons (Table 1). Component-level direct costs are presented in Supplementary Table S1. These estimates distinguish direct medical from direct non-medical expenditures and should be interpreted as gross pre-reimbursement amounts rather than net out-of-pocket costs.

### Factors associated with total disease burden

Table 2 presents the GLM for total disease burden.

**Table 2:** Generalized linear model of total disease burden.

Variable	Exp(β)	95% CI	*p*-value
Gender	1.046	0.744–1.469	0.796
Treatment Setting	0.126	0.095–0.168	<0.001
Age group (per category toward younger age)	0.856	0.765–0.959	0.007
Chronic Comorbidity	1.051	0.807–1.370	0.712
Rash Distribution	0.790	0.617–1.011	0.061
Rash Duration	1.251	0.975–1.607	0.078
PHN Status	0.511	0.387–0.674	<0.001

*N* = 222. Outcome: total burden. Exp(β) values were estimated using a GLM with a Gamma distribution and log link. Gender was coded as 1 = male and 2 = female, and the reported contrast is female vs. male. Age group was entered as a single ordinal variable coded 1 = ≥80 years, 2 = 70–79 years, 3 = 60–69 years, and 4 = 50–59 years; Exp(β) is the change per one-category step toward a younger age group, not a contrast with a reference category. Other categorical contrasts were outpatient vs. inpatient; no vs. yes chronic comorbidity; single vs. multiple dermatomes; rash duration ≥2 vs. <2 weeks; and no PHN vs. PHN. PHN, postherpetic neuralgia; CI, confidence interval.

After adjustment, treatment setting, PHN status, and age group were associated with total disease burden.

Outpatients had a lower expected total burden than inpatients (Exp(β) = 0.126, 95% CI 0.095–0.168; *p* < 0.001), and participants without PHN had a lower expected total burden than those with PHN (Exp(β) = 0.511, 95% CI 0.387–0.674; *p* < 0.001). Each one-category step toward a younger age group was associated with a 14.4% lower expected total burden (Exp(β) = 0.856, 95% CI 0.765–0.959; *p* = 0.007).

Chronic comorbidity, rash distribution, and rash duration were not significantly associated with total disease burden.

### Factors associated with direct disease burden

Table 3 presents the GLM for direct disease burden.

**Table 3:** Generalized linear model of direct disease burden.

Variable	Exp(β)	95% CI	*P*-value
Gender	1.096	0.767–1.566	0.613
Treatment Setting	0.120	0.089–0.162	<0.001
Age group (per category toward younger age)	0.856	0.766–0.958	0.007
Chronic Comorbidity	0.916	0.769–1.091	0.325
Rash Distribution	1.218	0.929–1.596	0.153
Rash Duration	1.200	0.919–1.568	0.181
PHN Status	0.516	0.383–0.696	<0.001

*N* = 222. Outcome: direct burden. Exp(β) values were estimated using a GLM with a Gamma distribution and log link. Gender was coded as 1 = male and 2 = female, and the reported contrast is female vs. male. Age group was entered as a single ordinal variable coded 1 = ≥80 years, 2 = 70–79 years, 3 = 60–69 years, and 4 = 50–59 years; Exp(β) is the change per one-category step toward a younger age group, not a contrast with a reference category. Other categorical contrasts were outpatient vs. inpatient; no vs. yes chronic comorbidity; single vs. multiple dermatomes; rash duration ≥2 vs. <2 weeks; and no PHN vs. PHN. PHN, postherpetic neuralgia; CI, confidence interval.

After adjustment, treatment setting, PHN status, and age group were associated with direct disease burden.

Outpatients had a lower expected direct burden than inpatients (Exp(β) = 0.120, 95% CI 0.089–0.162; *p* < 0.001), and participants without PHN had a lower expected direct burden than those with PHN (Exp(β) = 0.516, 95% CI 0.383–0.696; *p* < 0.001). Each one-category step toward a younger age group was associated with a 14.4% lower expected direct burden (Exp(β) = 0.856, 95% CI 0.766–0.958; *p* = 0.007).

Chronic comorbidity, rash distribution, and rash duration were not significantly associated with direct disease burden.

### Factors associated with indirect disease burden

Table 4 presents the GLM for indirect disease burden.

**Table 4:** Generalized linear model of indirect disease burden.

Variable	Exp(β)	95% CI	*P*-value
Gender	0.990	0.810–1.210	0.924
Treatment setting	0.517	0.424–0.631	<0.001
Age, years	1.011	1.003–1.019	0.005
Chronic comorbidity	1.030	0.918–1.157	0.611
Rash distribution	0.944	0.788–1.131	0.530
Rash duration	1.237	1.036–1.476	0.019
PHN Status	0.581	0.476–0.709	<0.001

*N* = 222. Outcome: indirect burden. Exp(β) values were estimated using a GLM with a Gamma distribution and log link. Gender was coded as 1 = male and 2 = female, with male (code 1) as the reference category. Reported categorical contrasts were outpatient vs. inpatient; no vs. yes chronic comorbidity; single vs. multiple dermatomes; rash duration ≥2 vs. <2 weeks; and no PHN vs. PHN. Age was entered as a continuous variable in years. PHN, postherpetic neuralgia; CI, confidence interval.

After adjustment, treatment setting, PHN status, rash duration, and age were associated with indirect disease burden.

Outpatients had a lower expected indirect burden than inpatients (Exp(β) = 0.517, 95% CI 0.424–0.631; *p* < 0.001), and participants without PHN had a lower expected indirect burden than those with PHN (Exp(β) = 0.581, 95% CI 0.476–0.709; *p* < 0.001).

A rash duration of at least 2 weeks was associated with higher indirect burden than a duration of less than 2 weeks (Exp(β) = 1.237, 95% CI 1.036–1.476; *p* = 0.019). Each additional year of age was associated with a 1.1% increase in expected indirect burden (Exp(β) = 1.011, 95% CI 1.003–1.019; *p* = 0.005).

Rash distribution and chronic comorbidity were not significantly associated with indirect disease burden.

### Health-related quality of life

Retrospectively reported HRQoL was low during the acute HZ phase. Among all 222 participants, the median EQ-5D-5L utility score was 0.40 (P25, P75: −0.03, 0.53), and the median EQ-VAS score was 10.00 (P25, P75: 10.00, 10.00).

Among the 62 participants who developed PHN, the median retrospectively reported PHN-phase EQ-5D-5L utility and EQ-VAS scores were 0.71 (P25, P75: 0.52, 0.84) and 60.00 (P25, P75: 50.00, 77.50), respectively. These phase-specific estimates should not be interpreted as improvement because the acute-phase column includes all 222 participants, whereas the PHN-phase column includes only the 62 participants with PHN.

Acute-phase EQ-5D-5L and EQ-VAS scores differed across age groups (*p* < 0.001 and *p* = 0.004, respectively). PHN-phase EQ-5D-5L and EQ-VAS scores also differed across age groups (*p* = 0.020 and *p* = 0.048, respectively), although the PHN-phase EQ-VAS medians did not show a monotonic age gradient (Table 5).

**Table 5:** Retrospectively reported health-related quality-of-life scores, by age group and phase.

Age group (years)	Acute HZ, *n*	Acute EQ-5D-5L, median (P25, P75)	Acute EQ-VAS, median (P25, P75)	PHN, *n*	PHN EQ-5D-5L, median (P25, P75)	PHN EQ-VAS, median (P25, P75)
50–59	58	0.54 (−0.12, 0.59)	10.00 (10.00, 10.00)	8	0.81 (0.77, 0.94)	60.00 (52.50, 85.00)
60–69	63	0.43 (0.17, 0.53)	10.00 (10.00, 27.50)	20	0.70 (0.56, 0.84)	70.00 (52.50, 77.50)
70–79	50	0.20 (−0.03, 0.46)	10.00 (10.00, 10.00)	12	0.68 (0.38, 0.77)	60.00 (50.00, 77.50)
≥80	51	0.04 (−0.15, 0.46)	10.00 (10.00, 10.00)	22	0.59 (0.39, 0.83)	70.00 (52.50, 77.50)
Overall	222	0.40 (−0.03, 0.53)	10.00 (10.00, 10.00)	62	0.71 (0.52, 0.84)	60.00 (50.00, 77.50)
*P*-value		<0.001	0.004		0.020	0.048

Values are presented as median (P25, P75). p values were calculated using the Kruskal–Wallis test. Acute HZ estimates were based on all participants with available acute-phase HRQoL data; PHN-phase estimates were based only on participants with PHN. HZ, herpes zoster; PHN, postherpetic neuralgia; EQ-5D-5L, EuroQol five-dimensional five-level questionnaire; EQ-VAS, EuroQol visual analog scale.

## Discussion

This study evaluated disease burden and HRQoL among adults aged 50 years or older with HZ in Huzhou, eastern China. Direct burden was the largest measured component of total burden, and participants with PHN or inpatient treatment experienced substantially higher burden than their counterparts. Retrospectively reported HRQoL was markedly impaired during both the acute HZ and PHN phases, with age-group differences within each phase. These findings provide regional evidence on HZ burden but should be interpreted in light of the cross-sectional design and retrospective assessment.

Direct burden, which combined direct medical and direct non-medical costs, represented the largest measured component of total burden (13, 21, 22, 25). Similar patterns have been reported in China, Japan, South Korea, and Europe (13, 21, 22, 36). The source-currency median direct burden was RMB 39,998.58 (P25, P75: RMB 11,792.27, 277,962.43), equivalent to USD 5,633.62 (P25, P75: USD 1,660.88, 39,149.64) after division by 7.1. This study measured gross HZ-attributable expenditures before reimbursement rather than net patient out-of-pocket payments. Comparisons with claims-based or post-reimbursement estimates should therefore account for this difference in cost definition. The high burden may also reflect the case mix: 70 participants (31.5%) received inpatient treatment, for whom the median HZ-attributable direct burden was RMB 315,960.01 (USD 44,501.41). This inpatient estimate was restricted to expenditures linked to the index HZ episode; unrelated medical expenditures were excluded. Component-level distributions are presented in Supplementary Table S1; aggregate findings are described using the broader term direct burden rather than direct medical costs alone.

PHN was one of the strongest correlates of disease burden in this study (10, 11, 17). Participants with PHN incurred higher direct, indirect, and total burdens than those without PHN, consistent with studies from Europe and Asia (10, 21, 36, 37). Persistent neuropathic pain may require repeated consultations, long-term pharmacological treatment, and additional healthcare resources (10, 21, 37). Although PHN can also affect families and caregivers (10, 38), the indirect-cost measure in this study included only the patient’s HZ-related days lost from paid work or usual productive activities and should not be interpreted as caregiver burden.

Inpatient treatment was another important correlate of higher burden (13, 21). Participants treated as inpatients had markedly higher direct, indirect, and total burdens than outpatients, consistent with reports from Europe and Asia (14, 15, 36). Inpatient treatment may proxy greater clinical severity and entails hospital stays, specialist consultations, examinations, and supportive treatment. Because this observational analysis cannot establish causality, these findings identify high-burden subgroups rather than the effect of hospitalization itself.

The ordinal age-group score was independently associated with total and direct burden, while continuous age in years was associated with indirect burden (2, 3, 12). Because the ordinal score was coded from the oldest group (1) to the youngest group (4), Exp(β) values below 1 indicate lower expected total and direct burden with each step toward a younger age group. The unadjusted estimates also increased across age groups in Table 1. Aging is accompanied by a decline in cell-mediated immunity, which increases susceptibility to varicella-zoster virus reactivation and contributes to a higher risk of PHN and other complications (6, 11). Kawai et al. (6) demonstrated that HZ and PHN incidence increase with advancing age. Older adults may require more healthcare resources and experience longer recovery periods than younger individuals (2). Given the rapid aging of the Chinese population, the burden attributable to HZ is expected to become increasingly important for healthcare systems.

Chronic comorbidity was not independently associated with disease burden after adjustment. Comorbid and immunocompromising conditions are recognized risk factors for HZ (39), but the present study combined diverse chronic conditions into a single variable. This classification, together with the sample size, may have reduced the ability to detect disease-specific economic effects.

Rash duration was associated with indirect but not direct burden. A prolonged disease course may lead to more patient time away from paid work or usual productive activities. The indirect-burden measure captured only the patient’s HZ-related time loss and did not quantify informal caregiving.

The study also documented substantial retrospectively reported impairment in HRQoL (8, 9, 17–19). The acute-phase median EQ-5D-5L utility and EQ-VAS scores were very low, indicating severe perceived impairment. In particular, the acute-phase EQ-VAS median of 10.00 is unusually low relative to many published HZ estimates and should be interpreted cautiously because the assessment was retrospective and may be influenced by recall or symptom anchoring (17–19, 40). Pain can affect mobility, sleep, emotional well-being, and social functioning (17, 40).

The phase-specific columns in Table 5 represent different analytic populations: acute-phase estimates include all 222 participants, whereas PHN-phase estimates include only the 62 participants with PHN. They therefore do not demonstrate within-person improvement. Acute-phase utility tended to be lower in older age groups, whereas patterns in the smaller PHN subgroup were not uniformly monotonic. A paired analysis restricted to participants with PHN is required before making any within-person comparison between phases.

The findings may inform, but do not directly establish, vaccination policy. Randomized trials have demonstrated high RZV efficacy against HZ and PHN among older adults (41, 42), and a claims-based cohort study has reported effectiveness in routine practice (43). An earlier report summarized economic considerations for RZV in older adults (44), whereas peer-reviewed economic evaluations have examined the cost-effectiveness of RZV in China (45–47). The present study did not measure vaccine effectiveness, uptake, or cost-effectiveness; it provides local burden estimates that may serve as inputs for future prevention and economic evaluations.

This study has several strengths. First, disease burden and HRQoL were evaluated simultaneously within the same study population. Second, direct costs, indirect costs, and PHN-related outcomes were assessed comprehensively. Third, healthcare expenditure data were verified using administrative records whenever available, which improved data accuracy.

Several limitations should be acknowledged. First, the cross-sectional design precludes causal inference. Second, HRQoL during the acute HZ and PHN phases was recalled retrospectively after completion of the acute phase; variable recall intervals may have introduced substantial recall bias. Third, although PHN status was confirmed through clinical records and interview, some clinical or coding misclassification cannot be excluded. Fourth, only 222 of 420 identified patients were analyzed, and individual-level characteristics of non-included patients were unavailable; therefore, non-response and selection bias cannot be excluded. Fifth, the single-city setting limits generalizability. Finally, pain severity was unavailable, and the small PHN sample limited phase- and age-specific analyses.

Overall, HZ was associated with substantial economic and health burden among adults aged 50 years or older in eastern China, particularly among those with PHN or inpatient treatment. These regional findings support continued evaluation of prevention strategies but do not themselves demonstrate vaccine effectiveness or cost-effectiveness.

## Conclusion

The HZ was associated with substantial disease burden and reduced retrospectively reported HRQoL among adults aged 50 years or older in Huzhou, China. PHN and inpatient treatment identified subgroups with particularly high burden. These findings may inform prevention policy and future evaluations of RZV, while the retrospective HRQoL assessment and incomplete participation warrant cautious interpretation.

## Data Availability

The original contributions presented in the study are included in the article/Supplementary material, further inquiries can be directed to the corresponding author.
