# Correction: Disease burden and health-related quality of life among adults aged 50 years or older with herpes zoster in Huzhou, China

**DOI:** 10.3389/fpubh.2026.2002685

**Published:** 2026-09-30

**Authors:** Shangyan Han, Jie Gong, Jianyong Shen, Xiaofu Luo, Zizhe Zhang, Xiaoqi Liu, Yu Hu

**Affiliations:** 1 School of Public Health, Hangzhou Medical College, Hangzhou, Zhejiang, China; 2 Institute of Immunization, Zhejiang Provincial Center for Disease Control and Prevention, Hangzhou, China; 3 Institute for Health Policy, Hangzhou Medical College, Hangzhou, Zhejiang, China; 4 Institute of Immunization, Huzhou Center for Disease Control and Prevention, Huzhou, China

**Keywords:** disease burden, EQ-5D-5L, EQ-VAS, health-related quality of life, HZ, older adults, PHN, vaccine

Affiliation “School of Public Health, Hangzhou Medical College, Hangzhou, Zhejiang, China” was omitted from the paper. Author Shangyan Han should have the affiliations “School of Public Health, Hangzhou Medical College, Hangzhou, Zhejiang, China” and “Institute of Immunization, Zhejiang Provincial Center for Disease Control and Prevention, Hangzhou, China”. The affiliations have been corrected for this author.

The province has been added to the affiliation “Institute for Health Policy, Hangzhou Medical College, Hangzhou, Zhejiang, China”.

The original version of this article has been updated.

