# Supplementary material for: Disease burden and health-related quality of life among adults aged 50 years or older with herpes zoster in Huzhou, China
Source: Front Public Health. 2026 Sep 9;14:1929296. doi: 10.3389/fpubh.2026.1929296 (PMC13625266; doi:10.3389/fpubh.2026.1929296)
Supplement: Supplementary file 1 [file Table_1.docx]

**Supplementary material**
**Supplementary Table S1. Component-level direct costs of herpes zoster among the study participants**

| **Cost component** | **Participants with non-zero cost, n (%)** | **Median cost among participants with non-zero cost, RMB (P25, P75)** | **USD equivalent, median**  **(P25, P75)** |
| --- | --- | --- | --- |
| **Direct medical costs** |  |  |  |
| Outpatient treatment | 152 (68.47) | 23,631.50(6,954.73, 48,802.49) | 3,328.38 (979.54, 6,873.59) |
| Inpatient treatment | 70 (31.53) | 315,960.01(94,743.75, 451,890.72) | 44,501.41 (13,344.19, 63,646.58) |
| Medicines | 50 (22.52) | 710.00 (220.23, 2,321.22) | 100.00 (31.02, 326.93) |
| **Direct non-medical costs** |  |  |  |
| Transportation | 222 (100.00) | 30.00 (10.00, 70.00) | 4.23 (1.41, 9.86) |
| Health products | 141 (63.51) | 79.00 (12.00, 374.00) | 11.13 (1.69, 52.68) |
| **Aggregate outcomes** |  |  |  |
| Direct medical costs | 222 (100.00) | 39,145.00 (10,952.75, 160,694.00) | 5,513.38 (1,542.64, 22,632.96) |
| Direct non-medical costs | 222 (100.00) | 30.00 (10.00, 70.00) | 4.23 (1.41, 9.86) |
| Direct burden | 222 (100.00) | 39,998.58 (11,792.27, 277,962.43) | 5,633.62 (1,660.88, 39,149.64) |

Note: The denominator is 222. Component values are among participants with non-zero costs. Direct medical costs are gross HZ-attributable amounts billed before reimbursement, not net patient out-of-pocket payments; USD = RMB/7.1. Direct medical costs include outpatient care, hospitalization and medicines. Direct non-medical costs include patient-paid transportation and health products. Direct burden = direct medical + direct non-medical costs.
